# Methicillin-Resistant *Staphylococcus aureus* among Students: Nasal Carriage Rate, Contributing Factors, and Antimicrobial Susceptibility

**DOI:** 10.3390/medicina60101590

**Published:** 2024-09-27

**Authors:** Ahsen Taqveem, Muhammad Hidayat Rasool, Bilal Aslam, Fatima Mujahid, Azalfah Ibrar, Hasan Ejaz, Abualgasim Elgaili Abdalla, Yasir Alruwaili, Muharib Alruwaili, Khalid Abosalif, Zeeshan Nawaz, Mohsin Khurshid

**Affiliations:** 1 Institute of Microbiology, Government College University Faisalabad, Faisalabad 38000, Pakistan; ahsentaqveem@gmail.com (A.T.); drmhrasool@gcuf.edu.pk (M.H.R.); drbilalaslam@gcuf.edu.pk (B.A.); fatimaajamal376@gmail.com (F.M.); azalfah.ibrar@gmail.com (A.I.); 2 Riphah College of Rehabilitation and Allied Health Science, Riphah International University, Faisalabad Campus, Faisalabad 44000, Pakistan; 3 Department of Clinical Laboratory Sciences, College of Applied Medical Sciences, Jouf University, Sakaka 72388, Saudi Arabia; hetariq@ju.edu.sa (H.E.); aealseddig@ju.edu.sa (A.E.A.); ysalruwaili@ju.edu.sa (Y.A.); mfalrwaili@ju.edu.sa (M.A.); koabosalif@ju.edu.sa (K.A.); 4 Sustainable Development Research and Innovation Center, Deanship of Graduate Studies and Scientific Research, Jouf University, Sakaka 72388, Saudi Arabia

**Keywords:** antimicrobial resistance, multi-drug resistant, public health, risk factors, *mecA* gene

## Abstract

*Background and Objectives*: *Staphylococcus aureus* is a prominent component of the human flora; however, it can cause various pathological conditions. The emergence of methicillin-resistant *S. aureus* (MR-SA) has been significantly influenced by the overuse and inappropriate administration of antibiotics. The frequency of MR-SA nasal colonization among healthcare workers (HCWs) is increasing, and MR-SA is not restricted to hospital settings, with a notable rise in infections among individuals unrelated to HCWs. This study aimed to assess the prevalence of *S. aureus* nasal carriage among students at Government College University Faisalabad (GCUF), University of Agriculture Faisalabad (UAF), a Government School (GS), and a Private School (PS) to characterize the phenotypic traits of isolates and evaluate antimicrobial resistance profiles. *Materials and Methods*: A total of 1200 nasal swabs were inoculated on blood and mannitol salt agar, followed by phenotypic identification of *S. aureus* and MR-SA using biochemical tests. Antimicrobial susceptibility testing was conducted via the Kirby–Bauer disk diffusion method, and minimum inhibitory concentration (MIC) determination was performed using the broth dilution method. Additionally, *nuc* and *mecA* gene amplification through PCR aided in isolate identification. *Results*: The results revealed that 14% (168) of students harbored *S. aureus* in their nasal cavities, with 8.5% (102) carrying methicillin-sensitive *S. aureus* (MSSA) and 5.5% (66) carrying MR-SA. Male students exhibited higher *S. aureus* (57.7%) and MR-SA (21.4%) prevalence compared to females (42.3% and 17.9%, respectively). Urban students showed a higher *S. aureus* prevalence (54.2%), while rural students exhibited a higher MR-SA rate (22%). Overall, 80.3% of *S. aureus* isolates displayed resistance to erythromycin followed by fluoroquinolones (47.6%) and clindamycin (42.2%). All the *S. aureus* isolates, including MR-SA, remained susceptible to vancomycin and linezolid. PCR results revealed that 95.5% (63) of MR-SA isolates carried the *mecA* gene. *Conclusions*: The high prevalence of multi-drug-resistant (MDR) *S. aureus* raises significant public health concerns, with educational institutions potentially serving as reservoirs for bacterial transmission. The improper use of antibiotics contributes to bacterial resistance and increased infection rates. It is crucial to implement measures to prevent antibiotic misuse and develop comprehensive strategies within educational settings to effectively combat *S. aureus* and MR-SA prevalence.

## 1. Introduction

*Staphylococcus aureus* is a common microorganism found in the human body and is associated with various pathological conditions. Its pathogenicity is multifaceted, encompassing resistance to antimicrobial drugs [1,2,3] and a high proficiency for biofilm formation [4]. Nasal colonization by this bacterium occurs in approximately 30% of the general population and can lead to infections such as cellulitis, endocarditis, impetigo, osteomyelitis, pneumonia, septicemia, and toxic shock syndrome (TSS) [5,6]. The nasal carriage of *S. aureus* is influenced by various risk and demographic factors, including age, gender, immune system competency, ethnicity, chronic diseases, living conditions, and behavioral patterns [7]. Individuals who encounter a colonized patient or household member are at an increased risk of becoming colonized themselves [8,9].

Antimicrobial resistance (AMR) presents a significant global challenge due to the rapid increase in infections caused by resistant microbes and the limited development of new antimicrobial medications [10,11]. The emergence of MDR strains of *S. aureus*, specifically MR-SA, has heightened concerns about the necessity for judicious antibiotic use to prevent the spread of these resistant strains [12]. Risk factors for MR-SA infection include the overuse of antimicrobial agents, hospitalization, close contact with infected or colonized individuals, and various medical conditions. Additional risk factors comprise diabetes, advanced age, the presence of indwelling devices, intravenous drug use, and immunocompromised states [13,14,15]. Understanding the modes of transmission, colonization persistence, and antimicrobial patterns is crucial for developing effective prevention and control measures.

Students in educational institutions, such as schools, colleges, and universities, frequently interact with peers who may be colonized by *S. aureus*. The sharing of personal items and suboptimal sanitary conditions within these institutions raise significant public health concerns. This is particularly alarming because schools and universities can serve as reservoirs for the transmission of resistant bacteria. Therefore, this study aimed to evaluate the prevalence of MR-SA nasal colonization among students and their resistant patterns to commonly prescribed antibiotics in the study region.

## 2. Materials and Methods

### 2.1. Study Design and Ethical Approval

A cross-sectional investigation was conducted from September 2023 to March 2024 and involved the collection of nasal swabs from students across various educational institutions in Faisalabad. Nasal swab samples were collected following a previously described protocol [16]. This study was approved by our Institutional Ethical Review Committee under letter No GCUF/ERC/23/161 dated 28 September 2023 and the sampling was carried out in accordance with the biosafety standards and international safety rules. To avoid bias and ensure transparency, the objectives of this study and informed consent were presented to the patients.

### 2.2. Sample Size and Study Area

A total of 1200 nasal swab samples were collected from students who consented to participate across various educational institutions. The samples were distributed as follows: 400 samples each from GCUF and UAF and 200 samples each from GS and PS. This study utilized a non-probability convenience sampling technique, where students were recruited based on their availability and willingness to participate. The inclusion criteria required students to be enrolled in the respective institutions and without any recent antibiotic use (within the past 30 days). No exclusion criteria based on gender or ethnicity were applied. The confidentiality and rights of all participants were strictly protected throughout this study.

### 2.3. Data Collection

During sample collection, each student was given a pre-designed structured questionnaire that included dichotomous questions. The questionnaire primarily aimed to collect data regarding personal information, demographic characteristics, and presumed risk factors such as housing and living conditions, contact with animals, and hospital stays [17].

### 2.4. Sample Collection

The nasal swab samples were collected from students using a sterile cotton swab by a trained medical staff after obtaining verbal consent from each participant. The purpose and objectives of this study were clearly elaborated to each participant before the collection of samples. After obtaining the sample, the swabs were placed into a transport medium, labeled properly, and shifted to the laboratory in an icebox for further processing [18].

### 2.5. Isolation and Identification

The nasal swabs were cultured on mannitol salt agar (MSA) and blood agar and then incubated at 36 °C for 24 h [19]. Following purity plating, Gram staining was used to identify Gram-positive cocci. A catalase test was then conducted to differentiate Gram-positive Staphylococci from Streptococci based on the presence of the catalase enzyme. Subsequently, a coagulase test was performed to identify *S. aureus* and distinguish it from other coagulase-negative Staphylococci.

For confirmation of *S. aureus*, the *nuc* gene, which is considered the gold standard for its identification, was subsequently amplified. DNA extraction was performed using FavorPrep^TM^ Genomic DNA Extraction Kit in accordance with the instructions provided by the manufacturer [20]. The amplification of the *nuc* gene was performed using the following primers: F: 5′-GCGATTGATGGTGATACGGTT-3′ and R: 5′-AGCCAAGCCTTGACGAACTAAAGC-3′. It followed a previously published protocol [21]. The amplified products were visualized using a UV transilluminator following gel electrophoresis on a 1.5% agarose gel stained with ethidium bromide. A 100 bp Plus DNA Ladder was used as a reference to estimate the sizes of the amplicons.

### 2.6. Antimicrobial Susceptibility Patterns

The Kirby–Bauer disk diffusion method was employed to perform antimicrobial susceptibility testing on Mueller–Hinton agar (MHA). *S. aureus* colonies were suspended in normal saline, and turbidity was adjusted to match the 0.5 McFarland standard. This bacterial suspension was inoculated evenly over the entire surface of MHA plates using a sterile cotton swab. The inoculated plates were allowed to dry for 5 min with the lid on. The following antibiotics were then aseptically applied to the MHA plates: cefoxitin 30 μg, fusidic acid 10 µg, erythromycin 15 µg, ciprofloxacin 5 µg, linezolid 30 µg, trimethoprim–sulfamethoxazole 25 µg, clindamycin 2 µg, and levofloxacin 5 µg. The diameter of the inhibition zones for each antibiotic was measured with a ruler and interpreted according to CLSI guidelines (2024). The isolates were categorized as MDR if they exhibited resistance to at least one agent in three or more antimicrobial classes.

To determine the MIC of vancomycin, the broth microdilution method was used with the freshly prepared Mueller–Hinton broth and antibiotic stock solutions as per CLSI 2024 guidelines. For quality control purposes, *Pseudomonas aeruginosa* (ATCC-27853) and *Escherichia coli* (ATCC-25922) were used, and the results were interpreted in accordance with the CLSI guidelines 2024.

### 2.7. Amplification of mecA Gene

The *mecA* gene was amplified using primers F: 5′-GTAGAAATGACTGAACGTCCGATAA-3′ and R: 5′-CCAATTCCACATTGTTTCGGTCTAA-3′ as described in a previous study [22]. The steps of PCR were followed according to the previously described procedure [22] with a few changes. First, initial denaturation was carried out at 92 °C for 1 min, followed by 32 cycles of denaturation at 95 °C for 1 min, annealing at 50 °C for 45 s, and extension at 72 °C for 60 s. Finally, the amplification step was set at 72 °C for 5 min. The resulting amplicons were visualized using a UV transilluminator after gel electrophoresis on a 1.5% agarose gel stained with ethidium bromide. A 100 bp Plus DNA Ladder was included as a reference for estimating the size of the amplified products.

### 2.8. Data Analysis

Data obtained from the experiments were entered into an Excel spreadsheet, and IBM SPSS Statistics version 27.0 was used to perform statistical analysis. The odds ratio was calculated to assess the strength of association between two factors, while the chi-square test was used to determine the relationship between students from different institutions and the prevalence of *S. aureus*. A *p*-value of less than 0.05 was considered statistically significant.

## 3. Results

### 3.1. Socio-Demographic Characteristics

Out of 1200 nasal swab samples, 60.3% (723) were collected from males and 39.8% (477) from females across various institutes. Of these samples, 14% (168) tested positive for S. aureus. Among the positive cases, the majority were males (57.7%, 97/168), while females accounted for 42.3% (72/168). Age group analysis revealed that 400/1200 (33.3%) students were between 12 and 17 years, with 58/168 (34.5%) carriers, while 800/1200 (66.7%) were aged 19 to 26, with 110/168 (65.5%) carriers. The OR of 1.064 (95% CI: 0.755–1.5) for age group indicates no significant difference in nasal carriage between these age groups. Regarding area of residence, 732/1200 (61%) students were from urban areas, with 91/168 (54.2%) carriers, while 468/1200 (39%) were from rural areas, with 77/168 (45.8%) carriers. The OR of 0.721 (95% CI: 0.519–1.001) suggests no significant association between the area of residence and nasal carriage (Table 1).

### 3.2. Risk Factor Distribution

Hospital exposure was reported by 419/1200 (34.9%) students, with 60/168 (35.7%) nasal carriers, and no significant association was found (OR: 0.960, 95% CI: 0.683–1.350). Similarly, smoking was noted in 227/1200 (18.9%) students, with 36/168 (21.4%) carriers, and was not significantly associated with nasal carriage (OR: 0.833, 95% CI: 0.558–1.243). Chronic disease, present in 182/1200 (15.2%) students, was associated with a reduced risk, with 46/168 (27.4%) carriers, showing a significant protective effect (OR: 0.396, 95% CI: 0.269–0.581). Livestock exposure, found in 455/1200 (37.9%) students, with 48/168 (28.6%) carriers, significantly increased the risk (OR: 1.628, 95% CI: 1.139–2.237). Recent antibiotic use was reported by 472/1200 (39.3%) students, with 55/168 (32.7%) carriers, but no significant association was observed (OR: 1.393, 95% CI: 0.986–1.968). Dormitory residence, reported by 564/1200 (47%) students, with 67/168 (39.9%) carriers, was significantly associated with an increased risk (OR: 1.4, 95% CI: 1.005–1.952). Sharing personal items was common among 791/1200 (65.9%) students, with 112/168 (66.7%) carriers, but showed no significant association with nasal carriage (OR: 0.962, 95% CI: 0.681–1.359), as depicted in Table 2.

### 3.3. MR-SA Nasal Carriage Rate among Students

The prevalence of MR-SA in the study population was 5.5% (66/1200). Among the 168 *S. aureus* isolates, 39.2% (66/168) were methicillin-resistant. Among *S. aureus* nasal carriers, the proportion of males with MR-SA was 37.1% (36/97), compared to 42.3% (30/71) in females. In terms of age distribution, 37.9% (22/58) of carriers were aged 12 to 17 years, while 40.0% (44/110) were aged 19 to 26 years. Regarding residential background, 31.9% (29/91) of carriers were from urban areas, and 48.1% (37/77) were from rural regions. The detailed prevalence of MR-SA is presented in Table 3. Statistically, no significant association was observed between MR-SA prevalence and socio-demographic factors, except for residential background (χ^2^: 4.580, *p*-value: 0.032).

**Figure 1:**
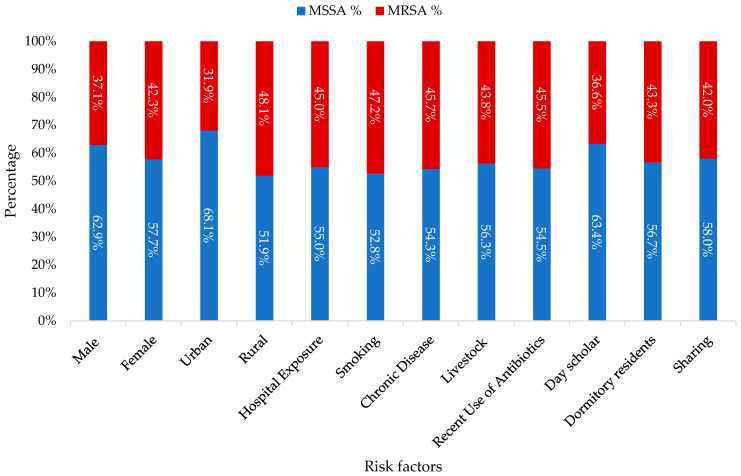
Comparison of MS-SA and MR-SA by demographic and risk factor data.

Among the 168 nasal carrier students, the majority (60.71%) tested positive for methicillin-susceptible *S. aureus* (MS-SA), while 39.28% were found to have MR-SA. An analysis comparing MS-SA and MR-SA across various demographic and risk factors is depicted in Figure 1.

The distribution of nasal carriers varied among different institutes. At GCUF, 28% (47) of students were carriers of *S. aureus*, with MS-SA accounting for 63.8% (30) and MR-SA for 36.2% (17). At UAF, 37.5% (63) of students tested positive for *S. aureus*, with MS-SA constituting 57.1% (36) and MR-SA 42.9% (27). In the PS, 15.5% (26) of students were found to be carriers of *S. aureus*, with 73.1% (19) being MS-SA and 26.9% (7) MR-SA. Finally, at the GS, 19% (32) of students carried *S. aureus*, with MS-SA at 53.1% (17) and MR-SA at 46.9% (15), as shown in Table 4.

### 3.4. Antimicrobial Susceptibility

All isolates were susceptible to vancomycin and linezolid (S = 100%, R = 0%). MR-SA exhibited 100% resistance to erythromycin, while the MS-SA isolates showed a resistance rate of 67.6% to erythromycin. The resistance patterns of isolates against different antibiotics are detailed in Table 5. The antimicrobial susceptibility and resistance patterns were quantified across various institutes, as presented in Table 6.

**Figure 2:**
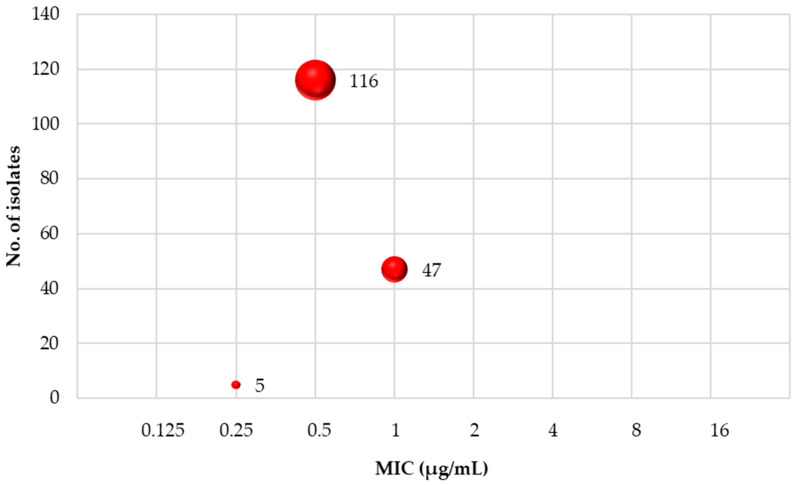
MIC distribution of vancomycin against *S. aureus* isolates.

The MIC of vancomycin for *S. aureus* was evaluated using a microtiter (96-well) plate, with results assessed either visually or by measuring optical density at 600 nm. All isolates were susceptible to vancomycin, with MIC values ranging between 0.25 and 1 µg/mL, as shown in Figure 2.

Of the 168 *S. aureus* isolates, 84 (50%) were identified as MDR. Among these, 50 isolates (59.6%) were resistant to three distinct antibiotic classes, while 8 isolates (9.5%) were resistant to six distinct classes of antibiotics (Table 7).

### 3.5. Distribution of mecA Genes

**Figure 3:**
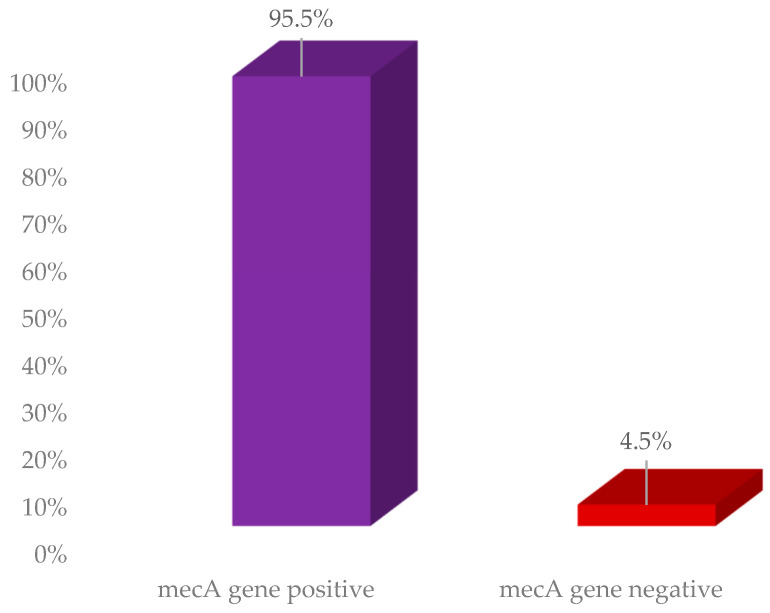
Prevalence of the *mecA* gene among MRSA isolates.

The presence of *mecA* genes was detected using PCR with specific primer sequences. Among the MR-SA isolates, *mecA* was detected in 95.5% (63), while 4.5% of MR-SA isolates did not possess this gene, as illustrated in Figure 3.

## 4. Discussion

The presence of *S. aureus* and MR-SA in the nasal cavity indeed establishes a significant risk factor for the development of various infections [23]. The emergence of MDR bacteria, particularly MR-SA, represents a major public health concern. It challenges conventional treatment strategies and infection control measures, necessitating enhanced surveillance and preventive measures.

According to this study, out of 1200 students evaluated, 168 (14%) were nasal carriers of *S. aureus*. This rate is lower than those reported in previous studies conducted in various regions of Pakistan, such as Rawalpindi (18.2%) [24], Lahore General hospital (23.42%) [25], and Khyber Pakhtunkhwa (20.5%) [26]. Furthermore, this study found a 5.5% prevalence rate of MR-SA (66 cases), which is lower than the rates reported in earlier studies conducted in Lahore Shaikh Zayed Hospital (10.7%) [27], Lahore General hospital (8.15%) [25], and Nishtar Medical University, Multan (9.3%) [28]. A lower prevalence rate of MR-SA was reported in Rawalpindi (1.5%) [24]. It is noteworthy that most studies in Pakistan have focused on healthcare workers, patients, and hospital environments, whereas our study examines the prevalence of *S. aureus* and MR-SA among students at different educational levels across various institutions. This approach offers a more comprehensive understanding of these bacteria within the student population.

Globally, the prevalence of *S. aureus* and MR-SA varies significantly across different geographical locations and populations. For example, studies have reported the following rates: Kabul (33.3% for *S. aureus* and 12.7% for MR-SA) [29], Nigeria (23% for *S. aureus* and 6% for MR-SA) [30], and Brazil (60% for *S. aureus* and 26.7% for MRSA) [31]. In contrast, our study observed a higher prevalence of MR-SA compared to other international studies, such as those in Malaysia (0%) [32], China (0.3%) [33], Tanzania (0.3%) [13], and Thailand (0%) [34]. The rates observed in our study are consistent with the global prevalence of chronic nasal carriage of *S. aureus*, which ranges from 12% to 30%. The variation in the prevalence of MR-SA and *S. aureus* among different studies can be attributed to differences in geographical regions, risk factors, and research methodologies.

The prevalence of *S. aureus* was observed to be higher among males compared to females, which is consistent with earlier findings showing 86.1% of male and 13.9% of female nasal carriers [35]. Although no statistically significant association (*p*-value = 0.5) was found between nasal colonization and male students in our study, it is plausible that behavioral factors among male students, such as hygiene practices, environmental exposure, and shared behaviors, could contribute to nasal colonization. Additionally, the disparity in participant numbers between males and females may have influenced these results. Male students participated more in this study compared to female students, likely due to the reluctance of females to provide samples in our society.

In contrast to previous studies that have linked MR-SA prevalence with various risk factors, such as hospital exposure, smoking, chronic disease, working with livestock, recent use of antibiotics, and shared behaviors, our study did not establish a statistical correlation between these factors and MR-SA colonization among students. For instance, a study reported that 27% of beef workers were carriers of *S. aureus*, but none had livestock-associated MR-SA [36]. Another study reported that smoking is an independent risk factor for MR-SA colonization [37].

The absence of an association between these risk factors and MR-SA colonization in the student population suggests that the frequency or intensity of these factors may not be sufficient to influence MR-SA colonization among students. Further research with larger sample sizes may be necessary to elucidate the relationship between these risk factors and MRSA colonization in this specific demographic. The results indicate a significant difference in the prevalence of MR-SA among students across various institutes, with higher rates observed at UAF and GS compared to GCUF and PS. Several factors could account for this variation in MR-SA prevalence across institutes, including differences in environmental conditions, hygiene standards, antibiotic use patterns, and demographic characteristics. Previous studies have underscored the impact of these factors on MR-SA prevalence in different populations. For instance, a study demonstrated that overcrowding and environmental conditions can contribute to higher rates of MR-SA colonization [38].

The isolates exhibited significant resistance to a broad range of commonly used first-line antibiotics in clinical practice. Higher levels of resistance were particularly evident for erythromycin, ciprofloxacin, levofloxacin, and clindamycin, which is consistent with previously reported results [39,40]. Notably, all isolates remained susceptible to vancomycin and linezolid, as documented in a previous study [28].

The analysis of antibiotic susceptibility patterns revealed that a higher proportion of MR-SA isolates exhibited resistance to multiple classes of antibiotics, indicating a significantly elevated level of antibiotic resistance in MR-SA (n = 60/66; 90.9%) compared to MS-SA (n = 24/102; 23.5%), consistent with the previous findings [35]. Resistance to trimethoprim-sulfamethoxazole, erythromycin, and ampicillin among *S. aureus* aligns with results from studies conducted in India [41], Tanzania [13], Ethiopia [42], Thailand [34], and Jima, Ethiopia [43]. This study was conducted in Faisalabad, Pakistan, where antimicrobial agents such as ampicillin, SXT, clindamycin, ciprofloxacin, and erythromycin are frequently used, potentially contributing to the emergence of antibiotic resistance.

Our study revealed a high prevalence (50%) of MDR *S. aureus*. Of these, 59% (50/85) were resistant to three distinct classes of antibiotics, with similar resistance patterns observed in Ethiopia [44]. However, our findings show a substantial disparity compared to a previously conducted study in Afghanistan [33]. This suggests that specific factors unique to each population may contribute to the development and spread of MDR *S. aureus*. Contributing factors could include differences in population demographics, risk factors, overcrowded wards, residential areas, and recent use of antibiotics across different countries. Moreover, the high prevalence of MDR *S. aureus* raises significant public health concerns, indicating that schools and universities could act as reservoirs for the transmission of resistant bacteria. This underscores the importance of implementing effective infection control measures and antibiotic stewardship programs in educational settings to mitigate the spread of multi-drug-resistant pathogens.

The current study identified three MR-SA isolates that tested negative for the *mecA* gene, which is consistent with findings from previous studies [45,46]. Subsequent investigation revealed that auxiliary genes [46], hyper-production of type-A-β-lactamase [45], modified normal PBPs [45], and *mecC* gene [46] were involved in conferring resistance to methicillin. This indicates that methicillin resistance is a dynamic and evolving phenomenon, with emerging strains exhibiting novel mechanisms of resistance beyond the traditional *mecA* gene.

One of the limitations of this study is the uneven distribution of the sample population, with a higher number of samples collected from male and older students compared to female and younger students. This imbalance may affect the generalizability of our findings. Cultural factors often lead to reluctance among females and younger students to participate in studies, which contributes to skewed sample distribution. Moreover, this study focused solely on the detection of the *mecA* gene due to resource constraints, whereas other *mec* gene variants, such as *mecC*, were not tested. Although these variants are less common, they may contribute to methicillin resistance. Comprehensive genotypic and phenotypic investigations are essential to elucidate the genetic pathways responsible for antibiotic resistance, which would be beneficial for future research. Consequently, further research within the community is necessary to determine the full extent of MR-SA prevalence and its implications.

## 5. Conclusions

Our findings underscore the importance of implementing comprehensive strategies within educational institutions to address the prevalence of *S. aureus* and MR-SA among students. The results highlight the need for the regular screening and monitoring of these infections in the nasal passages of individuals within educational settings. By routinely assessing the prevalence of *S. aureus* and MR-SA among the student population, educational institutions can gain valuable insights to inform targeted interventions. This proactive approach can not only protect the immediate community but also contribute to broader public health efforts aimed at mitigating the spread of antimicrobial-resistant pathogens.

## Figures and Tables

**Table 1:** Frequency distribution of socio-demographic characteristics and nasal carriage of *S. aureus* among students.

Demographic Data	Frequency (*n* = 1200) *n* (%)	Nasal Carriers (*n* = 168) *n* (%)	OR (CI of 95%)
Gender			
Male	723 (60.3)	97 (57.7)	0.886 (0.673–1.233)
Female	477 (39.8)	71 (42.3)	
Age group			
12 to 17	400 (33.3)	58 (34.5)	1.064 (0.755–1.5)
19 to 26	800 (66.7)	110 (65.5)	
Area			
Urban	732 (61)	91 (54.2)	0.721 (0.519–1.001)
Rural	468 (39)	77 (45.8)	

OR = odds ratio, CI = confidence interval.

**Table 2:** Frequency distribution of risk factors and nasal carriage of *S. aureus* among students.

Risk Factors	Frequency (*n* = 1200) *n* (%)	Nasal Carriers (*n* = 168) *n* (%)	OR (CI of 95%)
Hospital exposure			
Yes	419 (34.9)	60 (35.7)	0.960 (0.683–1.350)
No	781 (65.1)	108 (64.3)	
Smoking			
Yes	227 (18.9)	36 (21.4)	0.833 (0.558–1.243)
No	973 (81.1)	132 (78.6)	
Chronic disease			
Yes	182 (15.2)	46 (27.4)	0.396 (0.269–0.581)
No	1018 (84.8)	122 (72.6)	
Exposure to livestock			
Yes	455 (37.9)	48 (28.6)	1.628 (1.139–2.237)
No	745(62.1)	120 (71.4)	
Recent use of antibiotics			
Yes	472 (39.3)	55 (32.7)	1.393 (0.986–1.968)
No	728 (60.7)	113 (67.3)	
Residence			
Day scholar	636 (53)	101 (60.1)	1.4 (1.005–1.952)
Dormitory resident	564 (47)	67 (39.9)	
Sharing of personal items			
Yes	791 (65.9)	112 (66.7)	0.962 (0.681–1.359)
No	409 (34.1)	56 (33.3)	

OR = odds ratio, CI = confidence interval.

**Table 3:** Prevalence of MR-SA among nasal carrier students as assessed by chi-square analysis.

Demographic Data	*S. aureus* Nasal Carriage (Total = 168)	MR-SA (Total = 66)	X^2^ (*p*-Value)
	*n* (%)	*n* (%)	
Gender			
Male	97 (57.7)	36 (54.5)	0.454(0.5)
Female	71 (42.3)	30 (45.5)	
Age Group			
12 to 17	58 (34.5%)	22 (33.4)	0.068(0.794)
19 to 26	110 (65.5)	44 (66.6)	
Area			
Urban	91 (54.2)	29 (43.9)	4.580(0.032) *
Rural	77 (45.8)	37 (56.1)	
Hospital exposure	60 (35.7)	27 (40.9)	1.278(0.258)
Smoking	36 (21.4)	17 (25.7)	1.21(0.271)
Chronic disease	46 (27.4)	21 (31.8)	1.076(0.3)
Working with livestock	48 (28.6)	21 (31.8)	0.561(0.454)
Recent use of antibiotics	55 (32.7)	25 (37.8)	1.305(0.253)
Residence			
Day scholar	101 (60.1)	37 (56.1)	0.747(0.387)
Dormitory resident	67 (39.9)	29 (43.9)	
Sharing of personal use items	112 (66.7)	47 (71.2)	1.011(0.315)

* = statistically significant.

**Table 4:** Chi-square analysis of *S. aureus* distribution among students across various institutes.

Institutes	Total Isolates = 168		MS-SA		MR-SA	
	*n* (%)	*p*-Value	*n* (%)	*p*-Value	*n* (%)	*p*-Value
GCUF	47 (28)	0.3169	30 (63.8)	0.8254	17 (36.2)	0.1396
UAF	63 (37.5)		36 (57.1)		27 (42.9)	
PS	26 (15.5)		19 (73.1)		7 (26.9)	
GS	32 (19)		17 (53.1)		15 (46.9)	

**Table 5:** Antimicrobial resistance patterns of *S. aureus* isolates.

Antibiotics	MS-SA		MR-SA		Total	
	S *n* (%)	R *n* (%)	S *n* (%)	R *n* (%)	S *n* (%)	R *n* (%)
Cefoxitin	102 (100)	-	-	66 (100)	102 (60.7)	66 (39.2)
Ciprofloxacin	57 (55.9)	45 (44.1)	31 (47)	35 (53)	88 (52.4)	80 (47.6)
Levofloxacin	57 (55.9)	45 (44.1)	31 (47)	35 (53)	88 (52.4)	80 (47.6)
SXT	72 (70.6)	30 (29.4)	44 (66.7)	22 (33.3)	116 (69)	52 (30.9)
Fusidic acid	102 (100)	-	41 (62.1)	25 (37.9)	143 (85.1)	25 (14.8)
Vancomycin	102 (100)	-	66 (100)	-	168 (100)	-
Clindamycin	65 (63.7)	37 (36.3)	32 (48.5)	34 (51.5)	97 (57.7)	71 (42.2)
Erythromycin	33 (32.4)	69 (67.6)	-	66 (100)	33 (19.6)	135 (80.3)
Linezolid	102 (100)	-	66 (100)	-	168 (100)	-

SXT = trimethoprim–sulfamethoxazole.

**Table 6:** Institute-wise antimicrobial susceptibility pattern.

Antibiotics	GCUF (*n* = 47)		UAF (*n* = 63)		PS (*n* = 26)		GS (*n* = 32)	
	S *n* (%)	R *n* (%)	S *n* (%)	R *n* (%)	S *n* (%)	R *n* (%)	S *n* (%)	R *n* (%)
Cefoxitin	30 (63.8)	17 (36.2)	36 (57.1)	27 (42.9)	19 (73.1)	7 (26.9)	17 (53.1)	15 (46.9)
Ciprofloxacin	27 (57.4)	20 (42.6)	29 (46)	34 (54)	11 (42.3)	15 (57.7)	21 (65.6)	11 (34.4)
Levofloxacin	27 (57.4)	20 (42.6)	29 (46)	34 (54)	11 (42.3)	15 (57.7)	21 (65.6)	11 (34.4)
SXT	24 (51.1)	23 (48.9)	49 (77.8)	14 (22.2)	21 (80.8)	5 (19.2)	21 (65.6)	11 (34.4)
Fusidic acid	36 (76.6)	11 (23.4)	53 (84.1)	10 (15.9)	24 (92.3)	2 (7.7)	30 (93.8)	2 (6.3)
Vancomycin	47 (100)	-	63 (100)	-	26 (100)	-	32 (100)	-
Clindamycin	26 (55.3)	21 (44.7)	34 (54.0)	29 (46)	12 (46.2)	14 (53.8)	25 (78.1)	7 (21.9)
Erythromycin	7 (14.9)	40 (85.1)	14 (22.2)	49 (77.8)	7 (26.9)	19 (73.1)	5 (15.6)	27 (84.4)
Linezolid	47 (100)	-	63 (100)	-	26 (100)	-	32 (100)	-

SXT = trimethoprim–sulfamethoxazole.

**Table 7:** Antimicrobial agent-wise distribution of MDR *S. aureus*.

No. of Antibiotics	Antibiotic	Resistant Strains	
		Number	Percentage
3	CIP, DA, E	6	59.6
	CIP, SXT, DA	15	
	FOX, DA, E	17	
	FOX, CIP, E	12	
4	CIP, SXT, DA, E	3	20.2
	FOX, SXT, FD, E	8	
	FOX, CIP, SXT, E	6	
5	FOX, CIP, FD, DA, E	9	10.7
6	FOX, CIP, SXT, FD, DA, E	8	9.5

CIP, ciprofloxacin; DA, clindamycin; E, erythromycin; SXT, trimethoprim–sulfamethoxazole; FOX, cefoxitin; FD, fusidic acid.

## Data Availability

The original contributions presented in this study are included in the article; further inquiries can be directed to the corresponding authors.
